# Eating symptomatology and general psychopathology in patients with anorexia nervosa from China, UK and Spain: A cross-cultural study examining the role of social attitudes

**DOI:** 10.1371/journal.pone.0173781

**Published:** 2017-03-16

**Authors:** Zaida Agüera, Nicola Brewin, Jue Chen, Roser Granero, Qing Kang, Fernando Fernandez-Aranda, Jon Arcelus

**Affiliations:** 1 CIBER Fisiopatología Obesidad y Nutrición (CIBEROBN; CB06/03), Instituto Salud Carlos III, Barcelona, Spain; 2 Department of Psychiatry, University Hospital of Bellvitge-IDIBELL, Barcelona, Spain; 3 Leicestershire Eating Disorder Service, Bennion Centre, Leicester Glenfield Hospital, Leicester, United Kingdom; 4 Department of clinical Psychology, Shanghai Mental Health Center, Shanghai Jiao Tong University School of Medicine, Shanghai, PRC; 5 Departament de Psicobiologia i Metodologia. Universitat Autònoma de Barcelona, Barcelona, Spain; 6 Department of Clinical Sciences, School of Medicine, University of Barcelona, Barcelona, Spain; 7 Institute of Mental Health, Faculty of Medicine & Health Sciences, University of Nottingham, Nottingham, United Kingdom; Kyoto University, JAPAN

## Abstract

Cultural studies exploring differences in the manifestation of anorexia nervosa (AN) have primarily focus on Western and non-Western cultures. However, no study so far has considered the role that social attitudes (i.e. Collectivist vs. Individualist cultural values) have in the clinical manifestations of eating disorders, including AN patients. With this in mind, the aim of this study is to compare eating and general psychopathology in a large sample of individuals diagnosed with AN from China, Spain, and United Kingdom (UK), in order to study the differences according to belonging to Western or non-Western country, or the country's Individualist Index (IDV). The total sample comprised on 544 adults with a diagnosis of AN recruited from People´s Republic of China (n = 72), UK (n = 117), and Spain (n = 355). Assessment measures included the Eating Disorders Inventory and the Symptom Checklist-90-Revised. Our results show significant differences in most of the eating and psychopathological indices between the three countries. Patients from Western societies (Spain and UK) share more similarities regarding psychopathological expression of AN than the non-Western country (China). While Western countries show higher levels of body dissatisfaction, somatization and overall psychopathology, Chinese patients tend to deny or minimize depression, anxiety and other psychopathological symptoms. Besides, the IDV shows cultural differences in the interpersonal sensitivity scale, being AN patients from UK (the more individualistic society) who presented with higher levels of interpersonal sensitivity (i.e. discomfort during interpersonal interactions and more negative expectations concerning interpersonal behavior). In conclusion, our findings suggest that psychopathological expression of AN is better explained by Western/Eastern influence than by individualist/collectivist values. Although the diagnosis for the eating disorder may be the same, differences in the psychopathology comorbid to the eating disorders may suggest the need for treatments to be modified according to the culture.

## Introduction

Biological and environmental factors have been found to play an important role in the development of anorexia nervosa (AN) [1]. When studying the epidemiology of AN (incidence and prevalence in different cultures) the influence of socio-cultural factors need to be considered [2]. Until recently, socio-cultural theories have considered AN as “Western culture-bound syndromes” specific to Caucasian women from industrialized societies [3]. Recent studies have suggested that the Westernization of society (i.e. globalization and exposure to Western media in non-Western countries) contributes to the development of AN in non-Western cultures [4–7]. However, other studies have found the presence of AN in societies where there is an absence of Western influence [3].

In the last few years there has been a growing interest in the clinical presentation of AN across different non-Western societies. This has been reflected by an increased number of studies, describing the prevalence and manifestation of AN, particularly from African and Latin American/Hispanic groups [8] with few studies from Asian individuals [8]. These studies have been part of the debate regarding whether the clinical manifestation of AN differ across cultures [3]. As many of the cross-cultural studies analysing AN in non-Western societies have been carried out within Western countries [9] these studies have been inconclusive.

In spite of this, some studies have found that Chinese patients with AN report less body dissatisfaction, absence of fat phobia and different weight control behaviours than Western cultures [10,11], while others suggest a similar symptomatic profile to Western eating disorder (ED) patients [12]. An increase of fat phobia among Chinese ED patients in the last years has also been suggested [13]. The difference in the manifestation of symptomatology across cultures may not be unique to AN, as differences in the expression of psychopathology (such as depression or anxiety) have also been reported [14,15]. For example, Chinese people tend to present more self-control and emotional restraint and less externalization of their emotions [16–18]. Kleinman [19] reported that in Chinese societies the experience of depression is physical rather than psychological.

Cultures in all the above studies have been divided into Western or non-Western. However, the values and attitudes regarding the individual´s relation to their social group can also divide cultures into Collectivistic and Individualistic [20]. Collectivist cultures put emphasis on the individual´s behaviour for the whole group, focusing on cooperative tasks. Conversely, individualistic societies are characterized by an emphasis on what makes the individual distinct, focusing in competitive tasks [21–23]. According to the cultural dimensions considered by Hofstede [24] some Western countries, like Spain, will be closer to the Non-Western countries, like China, than to other Western countries, like the United Kingdom (UK). The individualism (vs. collectivism) index (IDV) classifies both China and Spain as collectivistic societies while Anglo-Saxon countries as individualistic cultures [20,24–26]. In fact, China is considered as one of the highest collectivist society (IDV: 20) whereas UK presents amongst the highest of the individualist scores (IDV: 89). Spain, by contrast, in comparison with the rest of the European countries (except for Portugal) is the highest collectivistic society (IDV: 51) [27]. Literature on individualism and collectivism provides a framework for exploring the association between cultural values and psychological processes or mental health [28]. Although we have not been able to find researches regarding IDV and ED, studies analyzing psychopathological traits, much of them co-morbid and/or related with EDs, found that individuals from collectivistic societies reported greater levels of social anxiety [29,30], lower self-esteem [17], maladaptive perfectionism (mainly high parental expectations) and depression symptoms [31]; but also less emotional expression or suppression of emotions in order to keep the group harmony [32,33], than those from individualistic countries. Likewise, some of the characteristics of collectivistic society, such as high levels of parental overprotection, have been found to be directly associated with eating pathology in both Chinese [34,35] and Spanish culture [36] and may influence the manifestation of these disorders. Besides, Katzman and Lee [37] have theorized that women in collectivistic societies which are in a changing cultural environment (i.e. modern exposure and experimentation with individualistic models) might develop an ED as a reaction to socio-cultural disconnection and transition.

With the aim of adding into the cultural debate this study will compare ED symptomatology, including co-morbid psychopathology, in a large sample of individuals diagnosed with AN from China, Spain, and UK. This will provide an opportunity to assess eating psychopathology for a set of cultures with different collectivist/individualist values as well as Western/Eastern orientations. Specifically, UK represents Western/high Individualist culture, China has been identified as Eastern/ high Collectivist, and Spain has been described as Western/ intermediate Collectivist [27]. Greater differences between Spain and UK would be indicative that collectivist attitudes play a more significant role in the clinical symptomatology and comorbid psychopathology of patients with AN. Alternatively, greater differences between Spain and China would suggest the primary role of the Eastern/Western division. As the big majority of patients with EDs attending the Chinese services present with AN, and other disorders are rare, the study has focused on patients with AN only.

The present study tries to overcome the limitations of the previous literature. Although some studies have investigated EDs among different cultures, most of them have used non-Western ethnic groups living in Western countries, and they may be biased by not taking into account the process of acculturation. To our knowledge, this is the first cross-cultural study assessing whether ED are more influenced across Western/Eastern or Collectivism/Individualism cultural attitudes. Thus, the findings of this study could provide useful information in the understanding of the manifestation of EDs, which may help the identification and prevention of these conditions. The knowledge of cross-cultural influences may help to design targeted interventions (e.g. if the collectivist values are more influential than Western/Eastern orientations, the patients of collectivist cultures probably would benefit more of group therapy, family therapy or family meetings).

The study’s aims are twofold: 1) to compare the clinical presentation, including co-morbid psychopathology, between patients with AN from Western (UK and Spain) and Non-Western (People´s Republic of China (PRC)) cultures, and 2) to compare the clinical presentation, including co-morbid psychopathology, between patients with AN from the UK, PRC and Spain in order study the differences according to the country's IDV.

Taking into account the existing literature, we expect to find psychopathological differences between groups. However, we hypothesized that these differences may not be due only to Western vs. Non-Western influence, but Collectivist/Individualist values may shape the psychopathology expression (e.g. the group wellbeing encourages the suppression of emotions and individual psychopathology in favor of the group in collectivist cultures).

## Materials and methods

### Participants

The total sample comprised 544 adults with AN recruited from PRC (n = 72), UK (n = 117), and Spain (n = 355). Experienced psychologists and psychiatrists diagnosed all participants according to the Diagnostic and Statistical Manual of Mental Disorders, 4th edition revised (DSM-IV-TR) [38]. The diagnoses were reanalyzed post hoc using the recent DSM-5 criteria [39] in order to include patients with AN according to the new criteria. The Ethics Committee of each institution mentioned below approved this study.

Every individual above the age of 18 years assessed at the different services explained below between the period of 2003–2012 who fulfilled inclusion criteria as per the DSM-5 of AN, was invited into the study.

#### PRC participant

The Chinese AN sample was composed by 72 patients, who were inpatients or outpatients and recruited consecutively from the Department of Clinical Psychology in Shanghai Mental Health Center from 2003 to 2012. The patients were diagnosed according to DSM-IV [38] by at least two senior psychiatrists. All patients included in the study were born in Mainland China, and completed the battery of questionnaires without missing data.

#### UK participants

The total sample with ED from UK comprised 117 AN patients, consecutive assessed at the Leicestershire Eating Disorders Service in the UK which is part of the Leicestershire Partnership NHS Trust. The service offers assessment and treatment to a population of 1 million living in the counties of Leicestershire and Rutland. Patients completed the battery of questionnaires as part of the assessment process. Patients are assessed over two appointments using the Clinical Eating Disorders Rating Instrument (CEDRI) [40]. This is a semi-structured investigator based interview that measures eating-related behaviours and attitudes in accordance with DSM-IV criteria. The tool has been shown to have good reliability and validity [41]. Patients attended the service between January 2003 and October 2012. Only patients who were born in the UK were included in the study. Nine patients were excluded from the overall sample because they were born outside the UK (2 from China, 6 Other South-Asian and 1 Mixed white & South-Asian).

#### Spanish participants

The Spanish sample with EDs was composed of 355 patients with AN consecutively assessed at the Eating Disorders Unit of the Department of Psychiatry at the University Hospital of Bellvitge, in Barcelona. Only white patients born in Spain were included in the study. In the overall sample of AN 13 patients were excluded as they were born in Latin America countries, 1 Asia, 5 Africa and 5 patients from other European countries.

### Procedure and assessment

All patients were subjected to a comprehensive clinical assessment by specialized clinical psychologists and psychiatrists. Weight and body mass index (BMI) on the day of assessment were measured for all patients. In addition, as a standard procedure of clinical assessment, all the participants completed the commonly applied questionnaires in the field of EDs, comprising the Eating Disorders Inventory (EDI) [42] and the Symptom Checklist-Revised (SCL-90-R) [43]. The questionnaires were administered individually and voluntarily before starting the treatment.

- *Eating Disorders Inventory (EDI)* [42]. This is a reliable and valid 64-item multidimensional self-report questionnaire that assesses 8 different cognitive and behavioral sub-scales, which measure typical characteristics for ED: Drive for Thinness, Bulimia, Body Dissatisfaction, Ineffectiveness, Perfectionism, Interpersonal Distrust, Interoceptive Awareness, and Maturity Fears. All of these scales are answered on a 6-point likert scale from *Always* to *Never*, and provide standardized subscale scores. This instrument was translated and validated in Chinese [44] and Spanish populations [45] with a good internal consistency.
- *Symptom Checklist-90- Revised (SCL-90-R)* [43]. This test contains 90 items and helps to measure 9 primary psychopathological dimensions, which are: Somatization, Obsession-Compulsion, Interpersonal Sensitivity, Depression, Anxiety, Hostility, Phobic Anxiety, Paranoid Ideation and Psychoticism. In addition, it includes three global indices, which are a global severity index (GSI), designed to measure overall psychological distress; a positive symptom distress index (PSDI), designed to measure the intensity of symptoms as well as a positive symptom total (PST), which conveys the array of self-reported symptoms. The Global Severity Index can be used as a summary of the test. This scale has been validated in Chinese [46,47] and Spanish [48] populations, obtaining a good mean internal consistency.

### Ethics statement

The followed procedures were agreed upon and approved by the Ethics Committee of our institutions (namely the Ethics Committee of Clinical Research at the University Hospital of Bellvitge, the Research Ethics Committee of Leicestershire Partnership NHS Trust, and the Ethics Committee of Shanghai Mental Health Center) in accordance with the Helsinki Declaration of 1975 as revised in 1983. Written signed informed consent was obtained from all participants from China and Spain. In the case of UK data, those are historical and therefore they were obtained retrospectively by the Ethics Committee of the hospital. So, although written consent of the participant is not available, we have the agreement form from the hospital indicating that clinical data can be used. All patients were adults; no minors were involved in this study.

### Statistical analysis

Analyses were carried out with Stata13.1 for Windows. Due the strong association between ED related measures with age, onset, duration and BMI, comparisons for EDI-2 and SCL-90R scales were adjusted by these covariates (through analysis of covariance–ANCOVA- procedures) to avoid the presence of confounding effects in the results. For the same, binary logistic regression models also adjusted by these potential confounding variables compared the presence of binges and vomits and the use of laxatives and diuretics between groups. Cohen’s-*d* coefficient measured effect size for proportions differences and mean differences (moderate effect size was considered for |*d*|>0.50 and high effect size for |*d*|>0.80). Bonferroni-Simes correction was employed to avoid increase in Type-I error due to multiple statistical comparisons.

## Results

### Socio-demographic characteristics

Table 1 includes the frequency distribution for gender and civil status in the three countries. No statistical differences emerged between groups with the big majority of patients attending services being single female.

**Table 1:** Sociodemographics.

	China; *n* = 72		UK; *n* = 117		Spain; *n* = 355				
	*n*	*Percent*	*n*	*Percent*	*n*	*Percent*	χ^2^	*df*	*p*
Gender; *% Female*	69	95.8%	110	94.0%	332	93.5%	0.56	2	.754
*Male*	3	4.2%	7	6.0%	23	6.5%			
Civil status; *% Single*	69	95.8%	97	82.9%	298	83.9%	9.84	4	.065
*Married*	3	4.2%	12	10.3%	43	12.1%			
*Divorced*	0	0%	8	6.8%	14	3.9%			

*Note*. SD: standard deviation.

### Comparison of clinical and psychopathology features between countries

Table 2 contains the results of the logistic regression and ANCOVA procedures (adjusted by the covariates considered in this study) comparing the clinical and eating symptomatology measures between Chinese, UK and Spanish AN patients. AN patients from UK and Spain were significantly older than Chinese AN patients. Statistical differences emerged for most of the symptomatological features. UK AN patients showed more bulimic symptoms than the Spanish ones (namely higher frequency of bingeing, vomiting and laxative use). They also showed higher scores on drive for thinness, than both Chinese and Spanish AN patients. However, Spanish AN patients presented the highest scores on body dissatisfaction compared with the other two groups.

**Table 2:** Comparison for clinical variables between countries (China, UK and Spain).

	Prevalences				Pairwise comparisons (logistic regression)					
	China	UK	Spain	Group	UK vs China		Spain vs China		UK vs Spain	
	*n* = 72	*n* = 117	*n* = 355	*p*	*OR*	*|d|*	*OR*	*|d|*	*OR*	*|d|*
Binges	36.1%	39.3%	16.9%	< .001*	1.18	0.07	4.12*	0.45	3.19*	**0.51**^†^
Vomits	37.5%	49.6%	26.2%	< .001*	1.61	0.25	2.16*	0.24	2.77*	**0.50**^†^
Laxatives	18.1%	42.7%	16.9%	< .001*	2.51*	**0.56**^†^	1.70	0.03	3.67*	**0.59**^†^
Diuretics	9.72%	0%	7.32%	.326	—	0.46	2.08	0.09	—	0.40
	Means				Pairwise comparisons (ANOVA/ANCOVA)					
	China	UK	Spain	*p*	*MD*	*|d|*	*MD*	*|d|*	*MD*	*|d|*
Age (years-old)	21.76	25.49	25.43	.003*	3.72*	**0.53**^†^	3.66*	**0.62**^†^	0.06	0.01
Onset (years-old)	18.13	16.63	19.65	.030*	1.50	0.40	1.52*	0.33	3.07*	**0.56**^†^
Duration (years)	3.36	2.32	5.55	< .001*	1.04	0.23	2.19*	**0.52**^†^	3.23*	**0.59**^†^
Body mass index	15.4	16.0	16.05	.004*	0.65*	0.35	0.69*	0.41	-0.05	0.03
EDI: Drive thinness	7.94	13.26	8.89	< .001*	5.32*	**0.86**^†^	0.94	0.14	4.38*	**0.66**^†^
EDI: Body dissatisfy.	4.07	3.67	10.71	< .001*	0.41	0.09	6.64*	**1.12**^†^	7.05*	**1.09**^†^
EDI: Bulimia	4.62	3.49	2.39	< .001*	1.13	0.20	2.23*	0.46	1.10*	0.24
SCL-90: Somatization	0.63	1.36	1.42	< .001*	0.73*	**0.95**^†^	0.79*	**0.98**^†^	0.06	0.07
SCL-90: Obses./comp.	1.17	1.82	1.56	< .001*	0.65*	**0.67**^†^	0.39*	0.42	0.26*	0.28
SCL-90: Inter.sensit.	1.32	2.26	1.71	< .001*	0.94*	**0.95**^†^	0.39*	0.41	0.55*	**0.56**^†^
SCL-90: Depressive	1.61	2.23	2.00	< .001*	0.62*	**0.60**^†^	0.39*	0.38	0.23	0.24
SCL-90: Anxiety	1.09	1.56	1.46	< .001*	0.47*	**0.50**^†^	0.37*	0.42	0.10	0.10
SCL-90: Hostility	1.08	1.18	1.23	.515	0.09	0.11	0.15	0.16	0.06	0.06
SCL-90: Phobic anx.	0.71	1.03	0.82	.072	0.32*	0.37	0.11	0.14	0.21	0.23
SCL-90: Paranoid	1.00	1.42	1.26	.017*	0.42*	0.48	0.26*	0.31	0.16	0.17
SCL-90: Psychotic	0.98	1.25	1.10	.109	0.28	0.33	0.12	0.16	0.15	0.18
SCL-90: GSI score	1.10	1.66	1.48	< .001*	0.56*	**0.71**^†^	0.38*	**0.51**^†^	0.18	0.23
SCL-90: PST score	44.44	60.07	57.72	< .001*	15.62*	**0.75**^†^	13.27*	**0.63**^†^	2.35	0.12
SCL-90: PSDI score	1.97	2.36	2.16	< .001*	0.39*	**0.60**^†^	0.18*	0.29	0.21*	0.33

GSI: global severity index; PSDI: positive symptom distress index; PST: positive symptom total

*Note*. *OR*: odds ratio. *MD*: mean difference (contrast).

*|d|*: Cohen’s-d coefficient.

*p-values* include Bonferroni-Simes correction for multiple comparison.

Comparisons for EDI-2 and SCL-90R scales were adjusted by age, onset, duration and BMI (ANCOVA).

*Bold: significant comparison (.05 level).

^†^Bold: moderate (*|d|*>0.50) to high (*|d|*>0.80) effect size.

Regarding psychopathology, Chinese patients with AN showed significantly lower scores than both UK and Spanish patients in many of psychopathological variables measured by the SCL-90R, namely somatization, global severity index, and positive symptom total scores. Chinese AN patients reported significantly less obsessive-compulsive, depression, anxiety and positive symptom distress index than AN patients from UK. Patients with AN from UK also reported significantly more interpersonal sensitivity than both Chinese and Spanish patients.

### Comparison of clinical and psychopathology features between Western and non-Western countries

Out of the 544 patients with AN studied, 472 were from a Western country and 72 from an Eastern one. Statistical differences (adjusted by the covariates of the study) were found between the two groups in most of the variables. This indicated that patients from the Western society presented with higher levels of body dissatisfaction and were significantly older than patients from non-Western society. Interestingly Western patients also presented with higher levels of co-morbid psychopathology, mainly higher levels of somatization, interpersonal sensitivity, and total psychopathology (see Table 3).

**Table 3:** Comparison for clinical variables between western vs. non-western countries, and according to the country's IDV.

	Western	Eastern				Indiv.	Collect.			
	*n* = 472	*n* = 72	*OR*	*p*	*|d|*	*n* = 117	*n* = 427	*OR*	*p*	*|d|*
Binges	22.5%	36.1%	2.98	.001*	0.30	39.3%	20.1%	2.50	.001*	0.43
Vomits	32.0%	37.5%	1.59	.103	0.12	49.6%	28.1%	2.93	< .001*	0.45
Laxatives	23.3%	18.1%	1.15	.695	0.13	42.7%	17.1%	3.83	< .001*	**0.58**^**†**^
Diuretics	5.5%	9.7%	2.63	.049*	0.16	0%	7.7%	—	—	0.41
	Means		*MD*	*p*	*|d|*	Means		*MD*	*p*	*|d|*
Age (years-old)	25.44	21.76	3.68	< .001*	**0.59**^**†**^	25.49	24.81	0.68	.385	0.08
Onset (years-old)	19.44	18.13	1.31	.073	0.28	16.63	19.38	2.75	.019*	**0.54**^**†**^
Duration (years)	4.84	3.36	1.49	.027*	0.34	2.32	5.15	2.83	< .001*	**0.53**^**†**^
Body mass index	16.04	15.35	0.68	.001*	0.39	16.00	15.93	0.07	.671	0.04
EDI: Drive thinness	9.81	7.93	1.88	.043*	0.29	13.27	8.71	4.56	< .001*	**0.69**^**†**^
EDI: Body dissatisfy.	9.23	4.10	5.13	< .001*	**0.85**^**†**^	3.71	9.48	5.77	< .001*	**0.89**^**†**^
EDI: Bulimia	2.62	4.62	2.00	.001*	0.40	3.48	2.80	0.67	.222	0.14
SCL-90: Somatization	1.41	0.63	0.78	< .001*	**0.97**^**†**^	1.36	1.28	0.08	.480	0.09
SCL-90: Obses./comp.	1.62	1.17	0.45	< .001*	0.47	1.82	1.49	0.33	.004*	0.35
SCL-90: Inter.sensit.	1.83	1.32	0.51	< .001*	**0.52**^**†**^	2.26	1.64	0.62	< .001*	**0.62**^**†**^
SCL-90: Depressive	2.05	1.61	0.44	.001*	0.42	2.23	1.93	0.30	.015*	0.30
SCL-90: Anxiety	1.48	1.09	0.39	.002*	0.44	1.56	1.39	0.17	.150	0.17
SCL-90: Hostility	1.22	1.08	0.13	.295	0.15	1.18	1.21	0.03	.791	0.03
SCL-90: Phobic anx.	0.86	0.71	0.16	.200	0.19	1.03	0.80	0.23	.034*	0.25
SCL-90: Paranoid	1.29	1.00	0.30	.013*	0.35	1.42	1.21	0.20	.058	0.23
SCL-90: Psychotic	1.14	0.98	0.16	.148	0.20	1.26	1.08	0.17	.075	0.21
SCL-90: GSI score	1.52	1.10	0.42	< .001*	**0.53**^**†**^	1.66	1.41	0.25	.011*	0.31
SCL-90: PST score	58.23	44.44	13.79	< .001*	**0.66**^**†**^	60.11	55.41	4.71	.064	0.23
SCL-90: PSDI score	2.20	1.97	0.23	.009*	0.35	2.36	2.13	0.24	.002*	0.38

*Note*. *OR*: odds ratio. *MD*: mean difference (contrast).

*|d|*: Cohen’s-d coefficient.

Western: United Kingdom and Spain. Eastern: China.

Indiv: individualistic model (United Kingdom). Collect: collectivistic model (China and Spain).

GSI: global severity index; PSDI: positive symptom distress index; PST: positive symptom total. *p-values* include Bonferroni-Simes correction for multiple comparison.

Comparisons for EDI-2 and SCL-90R scales were adjusted by age, onset, duration and BMI (ANCOVA).

*Bold: significant comparison (.05 level).

^†^Bold: moderate (*|d|*>0.50) to high (*|d|*>0.80) effect size.

### Comparison of clinical and psychopathology features according to the country's IDV

Table 3 also contains the results of the comparison between individualistic vs. collectivistic societies (adjusted by the covariates of the study). Individualist countries showed higher drive for thinness and less body dissatisfaction than the collectivist one. Moreover, participants from individualistic societies also presented with high interpersonal sensitivity (see Table 3).

## Discussion

The current study assessed clinical and comorbid psychopathological differences between patients with AN patients from China, UK and Spain. These three countries were chosen as they offer differences in the levels of individualism and collectivism as well as being Western and Eastern countries. According to the Individualism (vs. collectivism) Index, China and Spain can be classified as collectivistic societies while Anglo-Saxon countries, such as the UK, as individualistic cultures [20,24–26]. At the same time UK and Spain are Western countries while China is not. This study not only extend previous research comparing non-Western versus Western populations living in their respective countries [9], but also identifies whether abnormal eating patterns, anorexic cognitions and general psychopathology are more influenced by Western/Eastern or Collectivism/Individualism cultural attitudes.

The study found that people attending services in the different countries differed by age, with older people attending services in Western countries. This could be explained by the increase in pathological eating and body dissatisfaction among middle aged and older women reported in recent Western studies [49]. These societies have started to move away from AN being seen as illness affecting young people only [50,51]. However, this may not be the case in Non Western countries and stigma regarding access of older people to eating disorders services may still exist [52]. Non-Western societies may still see AN as an illness occurring only in young females [53,54].

In terms of eating psychopathology the study also found clear differences in the clinical presentation of EDs between the three countries. Western countries presented with higher levels of drive for thinness and body dissatisfaction, and Chinese patients appeared less clinically ill. These results are in line with other studies that have shown that female subjects from China were less likely to have fear of fatness or fat phobia [10], or score lower on restraint and weight concern subscales of Eating Disorder Examination (EDE) [55]. The high levels of body dissatisfaction among Western patients were mainly influenced by scores in the Spanish group. This may be less to do with Westernization or collectivist society and more influenced by the local culture of a coastal Mediterranean city. In line with this hypothesis, the study of Jaeger et al. [56] comparing 12 countries found the most extreme body dissatisfaction in northern Mediterranean countries, followed by other European countries, and finally non-Western countries [56]. Likewise, the high levels of drive for thinness in both Western and individualist societies were determined by the high scores of the UK patients. Although these results are not completely in accordance with previous studies arguing that the thin ideal is due to the Western influence [57], they would support the hypothesis that collectivist values could minimize the expression of eating symptoms in patients with AN, such as drive for thinness, to avoid the strain on the family that could cause the illness.

Regarding general psychopathology, the study found significant differences in most of the psychopathological indices between the three countries. Overall, Western countries presented with higher scores in comorbid psychopathology. Surprisingly, the IDV value only influenced the scores of interpersonal sensitivity scale. This dimension focuses on feelings of personal inadequacy and inferiority, particularly in comparison to others. Self-consciousness, uneasiness and marked discomfort during interpersonal interactions are hallmark features of interpersonal sensitivity [43]. Patients with AN from individualistic societies were found to present higher scores on interpersonal sensitivity (i.e. more negative expectations concerning interpersonal behavior) than those from collectivistic ones. This may be due to collectivistic societies being highly interdependent and concerned with interpersonal harmony, while individualistic ones being more independent societies. Patients from the Western society (UK + Spain) showed the highest scores of comorbid somatization, interpersonal sensitivity, and overall psychopathology. Our findings show that patients from both Spain and UK are more similar regarding psychopathology than those from China. As noted above, greater differences between Spain and China would suggest the primary role of the Western division. Therefore, these results suggest that psychopathology expression is better explained by belonging to Western society than for individualist/collectivist values. These results are in line with previous studies reporting how Chinese symptom patterns might differ from those found in the West, and how Chinese people tend to deny depression, anxiety or other psychopathological symptoms [16,58]. Denial or minimization of symptoms may be more common in Chinese patients with AN, as they are culturally encouraged to use denial and minimization to cope with conditions deemed taboo, particularly mental and terminal illnesses [59]. Our results are also in line with other previous studies that failed to find association between social anxiety and individualism/collectivism [60,29].

Patients from China presented with surprisingly low levels of psychopathology, including depression and anxiety. This could be explained by the fact that eating disorders services are relatively new in non-Western societies and patients with comorbid conditions may access other mental health services, leaving the more “pure” and less comorbid individuals, whose weight is substantially and dangerously low, accessing ED services. This may also have explained the differences in BMI between the three groups.

### Limitations and future directions

The present study should be evaluated within the context of several limitations. First, we relied on subjective self-report measures, which are vulnerable to social desirability. Moreover, psychopathology was measured by SCL-90R, a questionnaire which was created in the West, and may not accurately capture the type and range of symptoms and the extent of distress that Chinese are experiencing. According to the recent literature, Chinese may be reporting their distress experience differently depending on whether they are self-reporting on a symptom scale or they are disclosing to therapist during a face-to-face interview [61,62]. Second, this study is limited by a cross-sectional design, therefore without prospective studies, it remains difficult to distinguish state versus trait characteristics among those with AN. Finally, since there is strong evidence that personality traits play an important role in symptomatic profiles, maintenance and prognosis of EDs, futures studies should assess temperament and character traits of AN patients from collectivist and individualist societies.

It is important to highlight that cultures are difficult to define. It is never possible to guarantee that people living in a certain culture belong to it as their family, friends or work may belong to another culture. Cultures sometime are more related to families than to society. There will always be sub-cultures within a culture. For example, we will never know whether patients with AN attending Chinese services do really belong to the Chinese culture as this culture has changed very rapidly over the years. Chinese culture, particularly in big cities like Shanghai where this study takes place, has moved towards a more Western culture with their individualistic way of living. The same can be said about the Spanish culture where the role of the family, friends and society may still be important, but is also rapidly moving towards consumism and individualism.

In spite of these limitations, the current study also features several strengths. This study examined patients diagnosed with AN while others have rather investigated disordered eating symptoms or abnormal eating only. Furthermore, to our knowledge, this is the first cross-cultural study assessing whether ED are more influenced across Western/Eastern or Collectivist/Individualist cultural attitudes. In addition, most cross-cultural studies have been carried out within Western countries, and only few studies have been conducted in the country of origin of the ED patients [9].

In spite of the major difficulties of undertaking a cultural study in the field of eating disorders, this study provides information from a big number of patients with AN from different countries and different cultures. It shows that although the diagnosis may be the same the presentation of these people is different.

## Conclusions

In conclusion, our findings showed a clear pattern where Western influence, and not individualist values, best explains the psychopathological expression comorbid to the eating disorder according to countries. This can suggest that treatments targeting the comorbid psychopathology may need to be adapted to take cultural aspects into consideration as some evidenced based treatments may not be suitable for all cultures. When discussing treatments for EDs, culture and the adaptation to a specific culture should be considered.
